# Extraskeletal Osteosarcoma: Analysis of Outcome of a Rare Neoplasm

**DOI:** 10.1080/13577140020008084

**Published:** 2000-09

**Authors:** Martin D. McCarter, Jonathan J. Lewis, Cristina R. Antonescu, Murray F. Brennan

**Affiliations:** 1 Department of Surgery Memorial Sloan–Kettering Cancer Center 1275 York Avenue New York NY 10021 USA; 2 Department of Pathology Memorial Sloan–Kettering Cancer Center 1275 York Avenue New York NY 10021 USA

## Abstract

*Purpose.* Extraskeletal osteosarcoma represents an unusual soft-tissue sarcoma that historically is reported to carry an exceptionally poor prognosis.The objectives of this study were to use a prospectively gathered sarcoma database to test the prevailing clinical bias and more accurately describe the natural history, characterize the prognostic features, estimate survival and evaluate treatment strategies for this unusual sarcoma.

*Patients and methods.* From a large database of nearly 4000 sarcomas at a single institution, 15 patients with pathologically confirmed extraskeletal osteosarcoma were analysed.

*Results and discussion.* Extraskeletal osteosarcoma usually occurs as a large, deep, high-grade lesion in the lower extremity of older patients. Overall and disease-specific survival at 5 years was 50%, with a median follow-up of 35 months (range 3– 200 months). Use of adjuvant chemotherapy or radiation therapy did not appear to influence survival, but an effect may have been missed by the relatively low numbers in each group.When matched to a comparable group of patients with stage III extremity sarcomas, there was no significant difference in overall or disease-specific survival between groups. Treatment for extraskeletal osteosarcoma should follow established guidelines for treatment of soft-tissue sarcomas, with the decision regarding adjuvant therapy to be based on individual risk factors.


Sarcoma (2000) 4, 119–123

ORIGINAL ARTICLE

### Extraskeletal osteosarcoma: analysis of outcome of a rare neoplasm

MARTIN D. McCARTER,[1] JONATHAN J. LEWIS,[1] CRISTINA R. ANTONESCU[2] & MURRAY F. BRENNAN[1]

[1]*Department of Surgery and* [2]*Department of Pathology, Memorial Sloan–Kettering Cancer Center, 1275 York Avenue, New York, NY 10021, USA*

**Abstract**

*Purpose*. Extraskeletal osteosarcoma represents an unusual soft-tissue sarcoma that historically is reported to carry an exceptionally poor prognosis. The objectives of this study were to use a prospectively gathered sarcoma database to test the prevailing clinical bias and more accurately describe the natural history, characterize the prognostic features, estimate survival and evaluate treatment strategies for this unusual sarcoma.

*Patients and methods*. From a large database of nearly 4000 sarcomas at a single institution, 15 patients with pathologically confirmed extraskeletal osteosarcoma were analysed.

*Results and discussion*. Extraskeletal osteosarcoma usually occurs as a large, deep, high-grade lesion in the lower extremity of older patients. Overall and disease-specific survival at 5 years was 50%, with a median follow-up of 35 months (range 3–200 months). Use of adjuvant chemotherapy or radiation therapy did not appear to influence survival, but an effect may have been missed by the relatively low numbers in each group. When matched to a comparable group of patients with stage III extremity sarcomas, there was no significant difference in overall or disease-specific survival between groups. Treatment for extraskeletal osteosarcoma should follow established guidelines for treatment of soft-tissue sarcomas, with the decision regarding adjuvant therapy to be based on individual risk factors.

**Key words**: *osteosarcoma, extraskeletal sarcoma, prospective, outcome*

### Introduction

Extraskeletal osteosarcoma (ESOS) is a malignant mesenchymal neoplasm capable of osteoid, bone or chondroid matrix production, located in the soft tissues and without connection to the skeleton, as determined by radiographic and intra-operative examination.[1] As compared with osteosarcoma of bone, ESOS is rare and occurs infrequently in patients under 40 years of age. Although several studies have associated the diagnosis of ESOS with a poor prognosis with an estimated mortality between 70% and 80%, only limited clinical information exists regarding the natural history of this unusual sarcoma.[1–4] Most series reported to date represent retrospective reviews, spanning up to 73 years in length, with the largest series containing 88 patients and often lacking pathologic confirmation or adequate follow-up information.[5,6] Previous prospective studies of soft-tissue sarcomas have not analyzed extraskeletal osteosarcomas separately, as the number of cases remains small.[7]

The objective of this study was to analyze a cohort of patients, testing the prevailing clinical bias that ESOS behaves in a highly aggressive fashion. In addition, we sought more accurately to describe the natural history, characterize the prognostic features, estimate survival and evaluate treatment strategies for this unusual soft-tissue sarcoma.

### Patients and methods

From 1 July 1982 to 30 June 1999, 3969 patients over the age of 16 years with soft-tissue sarcomas were admitted for treatment to the Memorial Sloan–Kettering Cancer Center and followed prospectively. Of these patients, 15 had pathologically confirmed ESOS and form the basis of this review.

For the purposes of this study, ESOS was defined as a sarcoma of the soft tissues having no attachment to bone or periosteum and which has a histologic appearance indistinguishable from that of primary osteogenic sarcoma of bone. Microscopically, the diagnostic features include the presence of matrix production, such as neoplastic osteoid or bone, and a not infrequent association with malignant cartilage. As with skeletal osteogenic sarcoma, the tumor cells of ESOS resemble either fibrosarcoma, malignant

Correspondence to: Jonathan J. Lewis, MD, PhD, Department of Surgery, Memorial Sloan–Kettering Cancer Center, 1275 York Avenue, New York, NY 10021, USA. Tel: 212 639-2940; Fax: 212 794-5847; E-mail: lewisj@mskcc.org

1357-714Xprint/1369-1643online/00/030119-05 © 2000 Taylor & Francis Ltd

*DOI: 10.1080/13577140020008084*

fibrous histiocytoma (fibroblastic osteosarcoma), malignant osteoblasts (osteoblastic osteosarcoma) or chondroblasts (chondroblastic osteosarcoma). Cases with minimal neoplastic osteoid formation, indistinguishable from malignant fibrous histiocytoma with malignant osseous metaplasia, were excluded from the analysis.[8] Patients who had a prior history of osteogenic sarcoma of the bone were also excluded. ESOSs of visceral organs (such as breast, uterus, bladder and kidney) were not included in the study, as in many of these tumors the presence of an epithelial component, suggesting carcinosarcoma, could not be ruled out. Furthermore, dedifferentiated tumors, such as liposarcoma, with osteogenic features were not included. Some patients had pre-operative imaging (plain films, computed tomography (CT) scan or magnetic resonance imaging (MRI) that showed no bony attachments; however, the final determination ruling out bone involvement was based on operative and pathology reports.

Data analyzed included patient characteristics (age, gender), tumor characteristics (size, grade, depth, location, histologic subtypes, local and distant recurrence), treatment (operation, microscopic margins, chemotherapy, radiation therapy) and clinical status as of 30 June 1999.

Since the majority of the ESOSs identified in the database were high-grade, deep and large lesions of the extremity (i.e. stage III), a comparison group of all extremity stage III sarcomas was identified and analyzed in a similar fashion.[9] This group (357 cases of stage III extremity sarcomas from the same clinical database) was used as the comparison group for purposes of statistical analysis. Multivariate analysis of prognostic factors was conducted using the Cox proportional hazards model.[10] Disease-specific and overall survival was calculated from the date of entry into the database until death or last day of follow-up. Survival analysis was performed using the actuarial method of Kaplan and Meier and comparisons between groups were made by log-rank analysis.[11]

### Results

### *Patient and tumor characteristics*

Fifteen of 3969 patients diagnosed with sarcoma had pathologically confirmed ESOS (Fig. 1). The median age of these patients was 61 years, and two-thirds of them were male (Table 1).

All tumors were deep (below the investing fascia) in location and histologically high-grade, and all but one located in the lower extremity. Three tumors were less than 5 cm in greatest dimension and 12 were greater than 5 cm.

### *Treatment and outcomes*

All patients underwent an operation, one requiring an amputation and 14 receiving limb-sparing surgery. The microscopic margins were negative in 13 patients and unknown (unable to be verified from referring institution) in the remaining two patients. In addition to surgery, three patients received chemotherapy, two

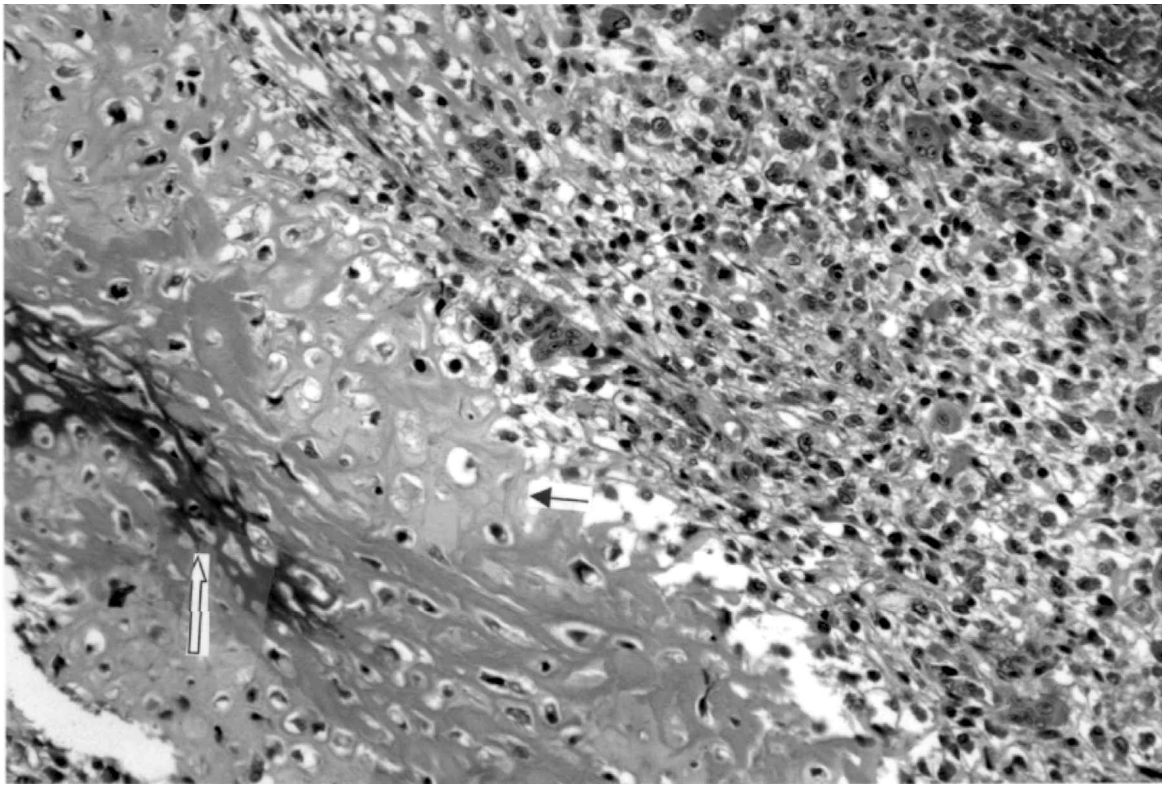

**Figure 1**. *Microscopic appearance of an ESOS showing neoplastic osteoid (large arrow) and focal cartilage matrix production (small arrow) by malignant stromal cells; histologic features otherwise indistinguishable from primary osteogenic sarcoma of bone.*

**Table 1**. *Summary of patient and tumor characteristics*

| ESOS characteristic ($N$=15) | |
|---|---|
| Age (years) | |
| Median | 61 |
| Range | 40–83 |
| Sex | |
| Male | 10 |
| Female | 5 |
| Site | |
| Lower extremity | 14 |
| Upper extremity | 1 |
| Size | |
| <5 cm | 3 |
| 5–10 cm | 5 |
| >10 cm | 7 |
| Grade | |
| High | 15 |
| Microscopic margin | |
| Positive | 0 |
| Negative | 13 |
| Unknown | 2 |

patients received some form of radiation therapy and three patients received a combination of both (Table 2).

The median follow-up for all patients was 35 months and for survivors was 28 months (range 3–200 months). Six patients recurred at distant sites (all in the lung) and one patient recurred both locally and in the lung. Seven patients have died of their disease, four are alive without evidence of disease, two are alive with disease and two died of other causes. Three of the four patients who are alive without disease received no adjuvant therapy.

The disease-specific and overall 5-year survival for patients with extraskeletal osteosarcoma is 50%. Table 3 shows a comparison between all patients with ESOS and a similar group of stage III extremity sarcoma patients selected to have large, deep and high-grade sarcomas. For reference purposes, similar adverse factors from a published report (using the same database) of all extremity sarcomas are also shown in the table. The difference in 5-year disease-specific survival (50% versus 38%) is not significantly different by log-rank analysis ($p$=0.4). This finding is better illustrated by the similar survival curves in Fig. 2. The relatively small number of patients in the ESOS group is reflected by the fact that a single death at 61 months results in a drop in disease-specific survival from 50% to 40%. Overall survival is likewise not significantly different between groups (curves not shown, $p$=0.35).

**Table 2**. *Treatment and outcomes*

| Option | $N$ |
|---|---|
| Surgery | |
| Limb-sparing | 14 |
| Amputation | 1 |
| Other therapy | |
| Chemotherapy | 3 |
| Radiation | 2 |
| Both | 3 |
| Recurrence | |
| Local and distant | 1 |
| Distant | 6 |
| Status | |
| Died of disease | 7 |
| Died of other disease | 2 |
| Alive with disease | 2 |
| No evidence of disease | 4 |
| Median survival (months) | 26 |
| Follow-up time (months) | |
| Mean | 53 |
| Median (all) | 35 |
| Median (survivors) | 28 |
| Range | 3–200 |

### Discussion

To our knowledge, this is the only analysis of patients with ESOS reported in the English literature that has been followed prospectively. Strict pathologic criteria were adhered to in categorizing the sarcoma as ESOS, ensuring that the presence of malignant osteoid formation was more than a focal process and that the tumor lacked connection with bone. Although there were larger series reported in the literature, these studies may suffer from their retrospective nature with a long look-back period and poor control over historical pathologic and clinical information.[2,5,12]

ESOS is an unusual malignant mesenchymal neoplasm, representing approximately 1% of all soft-tissue sarcomas.[13,14] In our large database spanning 17 years at the Memorial Sloan–Kettering Cancer Center, ESOS represents 0.04% of all sarcomas prospectively analyzed. The male to female ratio of 2:1 observed in this study is similar to that reported in some series; however, others have found no gender difference.[2,4,5,12] The median age of 61 years is considerably older than is generally reported in other retrospective series evaluating ESOSs and for all extremity sarcomas as a group.[1,3,7] The majority of these sarcomas were deep, large and high-grade in nature. Most reports also noted the predilection for these tumors to be deep-seated and large (mean size 8–10 cm) at the time of diagnosis. Plain radiographs occasionally revealed extraskeletal ossification, but are not a reliable means for detection or diagnosis. CT and MRI may be helpful but are not specific for diagnosing these tumors.

The current series found only one tumor in the upper extremity, none in the retroperitoneum and the remainder in the lower extremity. Most other series also demonstrated predominance of the lower extremity location, with the thigh being the most common subsite.[2,4]

ESOSs were generally treated according to the standard algorithm used for extremity sarcomas at the Memorial Sloan–Kettering Cancer Center. Limb-sparing surgery is used whenever possible, with the decision for adjuvant radiation or chemotherapy made on an individual basis depending on risk factors. In

**Table 3**. *Comparison of ESOS with other extremity soft-tissue sarcomas*

| | ESOS | Extremity stage III | Extremity all[7] |
|---|---|---|---|
| Number | 15 | 357 | 1041 |
| Age (years) | | | |
| Median | 61 | 54 | 51 |
| Range | 40–83 | 16–75 | 16–89 |
| Gender (%) | | | |
| Male | 67 | 60 | 53 |
| Female | 33 | 40 | 47 |
| Size (%) | | | |
| <5 cm | 20 | — | 41 |
| 5–10 cm | 33 | — | 28 |
| >10 cm | 47 | 100 | 25 |
| Grade (%) | | | |
| Low | — | — | 65 |
| High | 100 | 100 | 35 |
| Depth (%) | | | |
| Superficial | — | — | 24 |
| Deep | 100 | 100 | 76 |
| Site (%) | | | |
| Upper | 7 | 13 | 31 |
| Lower | 93 | 87 | 69 |
| Margin (%) | | | |
| Unknown | 13 | 15 | 2 |
| Positive | — | 24 | 23 |
| Negative | 87 | 61 | 75 |
| Recurrence (%) | | | |
| Local | 7 | 12 | 17 |
| Distant | 47 | 42 | 22 |
| Surgical treatment (%) | | | |
| Amputation | 7 | 11 | 10 |
| Limb-sparing | 93 | 75 | 87 |
| Adjuvant treatment (%) | | | |
| Chemotherapy | 40 | 49 | 23 |
| Radiation therapy | 33 | 56 | 40 |
| Median follow-up (months) | 35 | 31 | 48 |
| 5-year survival (%) | | | |
| Overall* | 50 | 33 | 76 |
| Disease-specific† | 50 | 38 | 81 |

(* $p$=0.35, †$p$=0.4)

general, adjuvant chemotherapy and radiation therapy are offered to patients with incomplete resections and to those with large (>5 cm) or high-grade lesions. There was only one local recurrence, and this patient recurred with both local and distant metastasis. As with other extremity sarcomas, distant metastasis was the most frequent cause of death.[7] Though the total numbers are small, the type of adjuvant therapy treatment employed did not appear to influence survival.

The overall poor survival reported in previous retrospective studies implied that ESOSs carry a worse prognosis than other sarcomas.[2,3,5,15] A recent study of all extremity sarcomas treated at the Memorial Sloan–Kettering Cancer Center reported an overall 5-year survival rate of 76% which is considerably better than the 50% 5-year survival rate for patients with ESOS.[7] In that study, patients with ESOS were combined with patients who had uncommon histologic subtypes. The group with uncommon histologic subtypes comprised 12% of all extremity sarcoma patients and had a 5-year disease-specific survival of 65%. A still more accurate comparison for this ESOS group would be with a comparable group of patients with deep, large and high-grade sarcomas of the extremity. As seen in the survival curves from Figs 2 and 3, there is no significant difference in overall or disease-specific survival when ESOSs are compared with a comparable group of stage III extremity sarcomas.

In conclusion, ESOS represents an unusual soft-tissue sarcoma that occurs as a large, deep and high-grade lesion of the extremities in older patients. The clinical and pathologic differential diagnosis includes myositis ossificans and malignant fibrous histiocytoma with focal bone metaplasia. Extraskeletal osteogenic sarcomas are grossly confined within the soft tissue, without attachment to the underlying bony structures. The pathologic diagnosis is confirmed by the presence of neoplastic osteoid, with or without cartilage matrix, produced by the malignant mesenchymal cells, identical morphologically to primary osteogenic sarcoma of the bone. Clinically, the prognosis for ESOS is similar to that for other

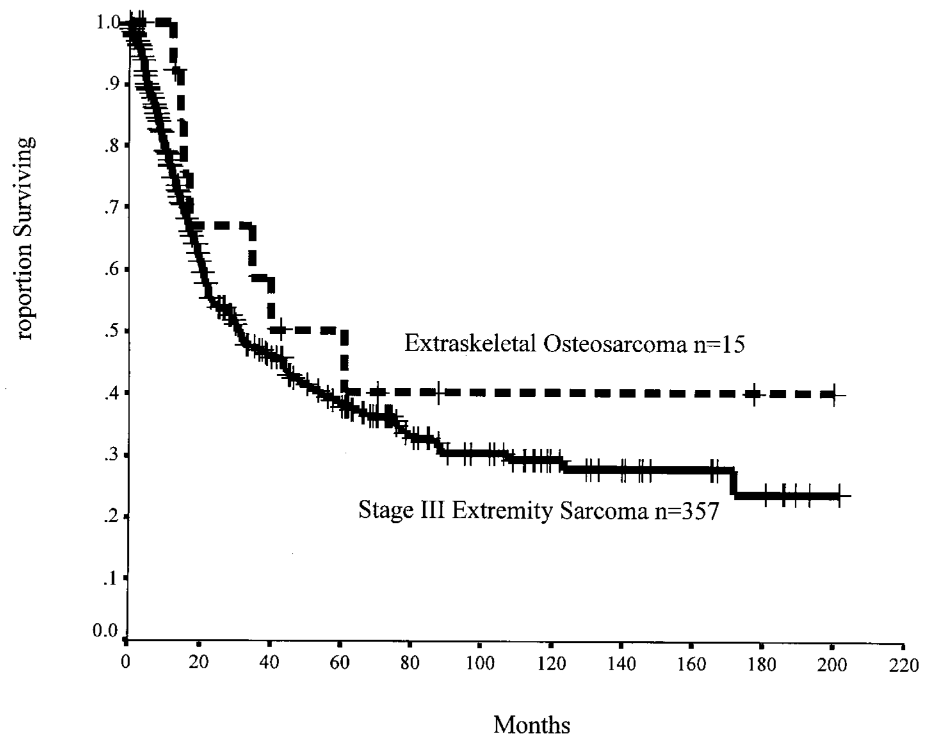

**Figure 2.** *Disease-specific survival for ESOS and stage III extremity sarcoma.*

comparably staged extremity sarcomas. Treatment decisions regarding adjuvant chemotherapy or radiation therapy for ESOS should be individualized, based on the assessment of relative risk factors. Such tumors need not be excluded from clinical trials, including soft-tissue sarcomas based on histology alone.

### Acknowledgement

This study was supported by grant CA-47179 (MFB).

### References

1 Enzinger FM, Weiss SW. *Soft tissue sarcomas.* St Louis: Mosby-Year Book, 1995.
2 Sordillo PP, Hajdu SI, Magill GB, Golbey RB. Extraosseous osteogenic sarcoma. A review of 48 patients. *Cancer* 1983;51:727–34.
3 Lane JM, Healey JH. Extraskeletal malignant tumors of bone. In: Raaf JH, ed. *Soft tissue sarcomas: diagnosis and treatment.* St Louis: Mosby-Year Book, 1993:261–9.
4 Lidang Jensen M, Schumacher B, Myhre Jensen O *et al.* Extraskeletal osteosarcomas: a clinicopathologic study of 25 cases. *Am J Surg Pathol* 1998;22:588–94.
5 Lee JS, Fetsch JF, Wasdhal DA *et al.* A review of 40 patients with extraskeletal osteosarcoma. *Cancer* 1995;76:2253–9.
6 Chung EB, Enzinger FM. Extraskeletal osteosarcoma. *Cancer* 1987;60:1132–42.
7 Pisters PW, Leung DH, Woodruff J *et al.* Analysis of prognostic factors in 1041 patients with localized soft tissue sarcomas of the extremities. *J Clin Oncol* 1996;14:1679–89.
8 Bhagavan BS, Dorfman HD. The significance of bone and cartilage formation in malignant fibrous histiocytoma of soft tissue. *Cancer* 1982;49:480–8.
9 Fleming ID, Cooper JS, Henson DE *et al. AJCC cancer staging manual—fifth edition.* Philadelphia: Lippincott-Raven, 1997:149–56.
10 Cox D. Regression models and life tables (with discussion). *J R Stat Soc B* 1972;34:187–220.
11 Kaplan EL, Meier P. Nonparametric estimation from incomplete observations. *J Am Stat Assoc* 1958;53:457–62.
12 Bane BL, Evans HL, Ro JY *et al.* Extraskeletal osteosarcoma. A clinicopathologic review of 26 cases. *Cancer* 1990;65:2762–70.
13 Allan CJ, Soule EH. Osteogenic sarcoma of the somatic soft tissues. Clinicopathologic study of 26 cases and review of literature. *Cancer* 1971;27:1121–33.
14 Lorentzon R, Larsson SE, Boquist L. Extra-osseous osteosarcoma: a clinical and histopathologic study of four cases. *J Bone Joint Surg* 1979;61B:205.
15 Lewis JJB, MB. Soft tissue sarcomas. In: Sabiston D, ed. *Textbook of surgery: the biologic basis of modern surgical practice.* Philadelphia: W. B. Saunders, 1997:528–34.

## References

[PDF_00401] Allan C. J., Soule E. H. (1971). Osteogenic sarcoma of the somatic soft tissues. Clinicopathologic study of 26 cases and review of literature.. Cancer.

[PDF_00398] Bane B. L., Evans H. L., Ro J. Y., Carrasco C. H., Grignon D. J., Benjamin R. S., Ayala A. G. (1990). Extraskeletal osteosarcoma. A clinicopathologic review of 26 cases.. Cancer.

[PDF_00387] Bhagavan B. S., Dorfman H. D. (1982). The significance of bone and cartilage formation in malignant fibrous histiocytoma of soft tissue.. Cancer.

[PDF_00381] Chung E. B., Enzinger F. M. (1987). Extraskeletal osteosarcoma.. Cancer.

[PDF_00378] Lee J. S., Fetsch J. F., Wasdhal D. A., Lee B. P., Pritchard D. J., Nascimento A. G. (1995). A review of 40 patients with extraskeletal osteosarcoma.. Cancer.

[PDF_00375] Lidang Jensen M., Schumacher B., Myhre Jensen O., Steen Nielsen O., Keller J. (1998). Extraskeletal osteosarcomas: a clinicopathologic study of 25 cases.. Am J Surg Pathol.

[PDF_00404] Lorentzon R., Larsson S. E., Boquist L. (1979). Extra-osseous osteosarcoma: a clinical and histopathological study of four cases.. J Bone Joint Surg Br.

[PDF_00383] Pisters P. W., Leung D. H., Woodruff J., Shi W., Brennan M. F. (1996). Analysis of prognostic factors in 1,041 patients with localized soft tissue sarcomas of the extremities.. J Clin Oncol.

[PDF_00369] Sordillo P. P., Hajdu S. I., Magill G. B., Golbey R. B. (1983). Extraosseous osteogenic sarcoma. A review of 48 patients.. Cancer.

